# Connecting care competencies and culture during disasters

**DOI:** 10.4103/0974-2700.50743

**Published:** 2009

**Authors:** Vivek Chhabra

**Affiliations:** National Disaster Management Authority (NDMA), Government of India, NDMA Bhavan, A-1, Safdarjung Enclave, New Delhi - 110029, India

**Keywords:** Disasters, care, competencies, culture

## Abstract

Connecting care Competencies and Culture are core fundamentals in responding to disasters. Thick coordination between professionals, communities and agencies in different geographical areas is crucial to the happening of appropriate preparedness and thus efficient response and mitigation of a disaster. In the next few articles, we present diverse examples related to the preparedness and recovery process to adverse disasters across the globe

## INTRODUCTION

Humans have evolved as the supreme species on the Earth, mainly because of the spirit and attitude they carry for supporting fellow brethren, especially in difficult times like disasters. The assistance, support and relief provided to the victims of a disaster by local and distant communities across the globe have always been overwhelming, continuous and repetitive. Besides providing relief and succor to the needy, these goodwill measures have now generated a knowledge-pool and understanding to build up frameworks for better preparedness in case of future catastrophes. In this issue of JETS, four disaster related topics have been discussed giving some important carry home messages.

## SWINE INFLUENZA VIRUS (SIV) A (H1N1) IN HUMANS

The people of our world are now facing the threat of the Influenza virus type A (H1N1) Infection which is normally found in pigs. We have no immunity and no vaccine to protect against this infection. The virus has been detected in Mexico and many other nations. Even though it has not been officially declared a pandemic the sheer potential of this virus to spread across the world calls for immediate action which includes strategies to isolate patients, protect the healthy and prevent the exposed with antiviral drugs. An exhaustive review of available current literature is presented by the authors.

## TEAM SINGAPORE AND CYCLONE NARGIS IN MYANMAR

The experiences of Team Singapore in providing humanitarian aid in the Cyclone *Nargis* affected areas in Philippines, signifies the importance of trained human resources, which unite for a common cause and perform effectively despite all the barriers. The article discusses the unique contingency plans implemented by the local administration, suiting the local needs besides discussing the issues of water, sanitation and hygiene along with health problems encountered. It was good to realize that psychological first-aid was considered and provided to the affected community and appropriate measures regarding essential protective and supportive measures to the aid providers were taken as well. This consolidates the new beginning of raising qualities and standards of care on the Asian landscapes.

## MODELS OF DISASTER RESPONSE FOR HOSPITALS

The article on joint triage model for multihospital mass casualty response emphasizes the importance of planning for interhospital coordination in response to disasters. Working together to merge resources is a very important step in the process of recovery post disasters.

## ISRAELI PERSPECTIVE ON RADIOLOGICAL TERRORISM

With the fear of terror attacks looming large, radiological terrorism is the latest threat to world peace. The article regarding health implications of radiological terrorism is very informative and emphasizes the importance of basic efficient medical care to bring down the mortality rates in a factual manner. The importance of tabletop and real life drills is well commented upon giving the due importance to the preparedness aspect.

## SELECTIVE COMPONENTS OF DISASTER MITIGATION

The role of a public health system is crucial in a disaster. It is well known that a disaster not only challenges the public health system but also holds potential to damage it. In the Indonesian Disasters of 2004 the important lesson learnt was, to deliver post-disaster public health in an equitable manner.

Analyzing the damages to the health sector, chalking out the needs of the damaged health sector, defining the role of existing public health structure to participate in the response,at the same time making itself recover are important aspects of the stabilization process. It is important to remember that post-disaster population is a vulnerable group.[1]

Another important component of disaster preparedness is having an information technology based database of information about disaster response personnel, equipment and agencies. Creating and sustaining such a facility is the joint responsibility of the government and the community, which is a perfect example of connecting to make a difference. The mere existence of such an initiative creates awareness and mobilizes the community to be better informed and prepared for contingencies.[2]

The people of the community struck by a disaster play a major role in the disaster mitigation process. One such example is the role of librarians in Disaster Response. It was discovered, by an oral history research project, that librarians played a major role in tracking the training of emergency experts, providing information, and participating in community as well as institution rebuilding efforts, during Hurricane Katrina, at the Oklahoma City.

Bombing and the SARS epidemic of 2003: Librarians played a major role in disaster mitigation at various levels of the government.[3]

Vulnerable groups like females, children, elderly, physically or mentally challenged are the worst sufferers during disaster due to inability to compete with others for relief material being provided in post-disaster phase.

Very vulnerable populations like the geriatric population - their age in combination with physiological, sensory, and cognitive changes make them have needs which require specialized care. Preparation, coordination and coalition between public health, disaster response teams, physicians and families is a important part of responding to their needs after a disaster. There is a need for professionals from different fields to work together to create community-wide disaster response plans.[4]

## INDIA AND DISASTER MITIGATION

The geo-climatic conditions of India play a significant role in increasing susceptibility to a natural as well as man-made disasters. More than 50% of Indian land is prone to earthquakes of moderate to very high intensity while 12 % of land is prone to floods and river erosion besides the 80% of coastline which is prone to cyclones and tsunamis. Compared to other parts of the globe, India faces a high vulnerability to Chemical, Biological, Radiological, Nuclear, and Explosive (CBRNE) attacks in view of her unique geographical location.[5]

In the last few decades, major natural catstrophes have hit India in form of 1999 Super Cyclone in Orissa which caused over 9000 deaths, the 2001 Bhuj earthquake which led to 14000 deaths, and the 2004 Tsunami which left behind 15,000 dead on the east coast of India. The man-made disaster in the form of Bhopal Gas Tragedy of 1984 has also shown its real impact by causing more than 15,000 deaths over the last 25 years.[6]

Like other developing nations, India is more prone to complex disasters. Hence, India took the step of institutionalizing disaster management 4 years back when the Disaster Management Act, 2005 was enacted.[7] This led to a paradigm shift from the relief centric approach to a complete and synergised approach which had prevention, mitigation and preparedness as the new core elements.

The holistic approach to disaster management is now being brought into practice in India. It has three elements in the pre-disaster phase i.e. Prevention, Mitigation and Preparedness, and three elements in post-disaster phase i.e. Response, Rehabilitation and Reconstruction[8]

**Figure 1:**
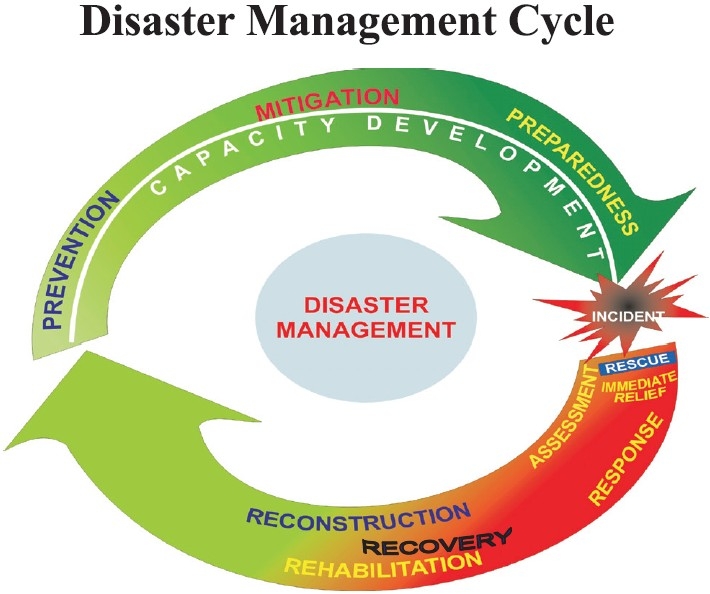
Disaster management – The holistic approach (SO: www.ndma.gov.in)

A time-bound approach was implemented by the Indian Government to transform the above thought-process into policy and plans. This was done by forming various core groups and steering committees with the help of various institutions, central ministries, states and other stakeholders at all levels. The resultant output was sprouting of the policies and guidelines following the various brainstorming consultations of the members of these groups[Figure 1].[8]

The framework and policies thus generated are in sync with the International Strategy for Disaster Reduction, the Rio Declaration, the Millennium Development Goals and the Hyogo Framework 2005-2015. The important areas covered include community-based disaster management, capacity development, consolidation of previous initiatives and best practices, cooperation with various stakeholders at all the levels thus generating a multi-sectoral synergy.[8]

**Figure 2:**
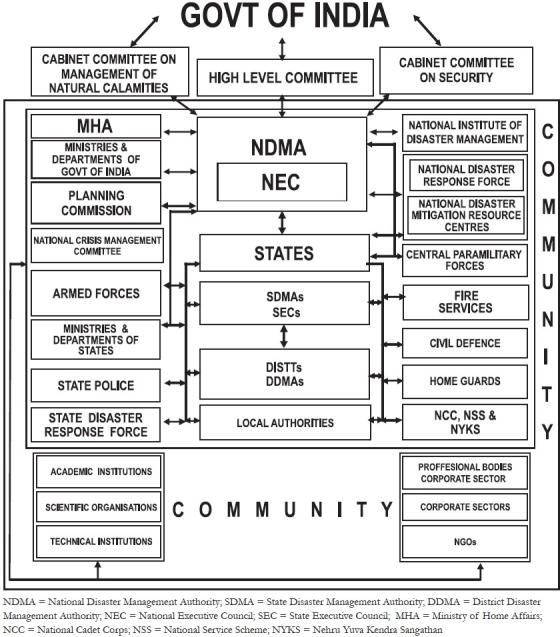
Synergies and interactive linkages in disater management (SO: ndma.gov.in)

The synergized management of disasters in India is reflected in the [Figure 2], which reflects interactive linkages at various functional levels, to optimize efficiencies.[8]

## NATIONAL DISASTER MANAGEMENT AUTHORITY

National Disaster Management Authority (NDMA) is an apex body of the Government of India which lays down policies and plans to ensure effective response to disasters. NDMA supports the National Executive Council (NEC), National Institute Disaster Management (NIDM), and eight battalions of National Disaster Response Force (NDRF). There is a State Level Disaster Management Authority (SDMA) which works with the State Disaster Response Force (SDRF) and State Executive Council (SEC). There is also a District Disaster Management Authority (DDMA) which works at the District Level. The Community remains a very crucial factor for the effective outcomes during the disasters.

## FUNCTIONS OF NDMA[8]

The multiple responsibilities undertaken by NDMA, Government of India helps in designing effective disaster management strategies well in advance. These tasks involve approving the national plan and getting consent to plans prepared by the ministries or government departments in accordance with the national plan. The various guidelines laid down over the last three years are now being followed by the state authorities in drawing up the state plans and by the different ministries or departments of the Government of India for disaster prevention or mitigation of its effects. The NDMA also manages the implementation of the policy and plan for disaster management, thus taking necessary steps for the prevention of disaster, mitigation, preparedness and capacity building. It has been authorized to recommend funds for mitigation besides making policies and guidelines for the functioning of the National Institute of Disaster Management (NIDM). The role of the NDMA spreads beyond the boundaries of India in the form of providing relief measures to other disaster affected countries subject to approval by the Central Government of India.

## ANALYSIS

Given the multicultural, socio-religious-linguistic fabric of the Indian society, we face the unique challenge of developing a common language and attitude towards a uniform, robust response in disaster prevention, preparedness and mitigation besides rehabilitation and reconstruction. The promulgation of activities of the disaster management structure in India is expected to build up efficiencies on the lines of airline business models and thus help the disaster managers navigate successfully with miniscule error rates during the worst times, saving precious lives, limbs and property. The above model may be replicated or modified, according to the regional needs, to strengthen the already existing disaster management structures of different parts of the world.

## CONCLUSION

Disasters will continue, but the need of the hour is common ways of communication during disasters. This can be developed from the airline industry where the pilots speak one common language in different parts of the world.

We as citizens of this one world can achieve a uniform code of conduct to deal with disasters. This is possible when experts, organizations and countries bridge their gaps and build networks to extrapolate their strengths to achieve a disaster resilient world.

## References

[1] Leitmann J (2007). Cities and calamities: learning from post-disaster response in Indonesia. J Urban Health.

[2] Troy DA, Carson A, Vanderbeek J, Hutton A (2008). Enhancing community-based disaster preparedness with information technology. Disasters.

[3] Featherstone RM, Lyon BJ, Ruffin AB (2008). Library roles in disaster response: an oral history project by the National Library of Medicine. J Med Libr Assoc.

[4] Aldrich N, Benson WF (2008). Disaster preparedness and the chronic disease needs of vulnerable older adults. Prev Chronic Dis.

[5] (2007). National Disaster Management Guidelines. Preparation of State Disaster Management Plans.

[6] (2007). National Disaster Management Guidelines. Medical Preparedness and Mass Casualty Management.

[7] (2007). National Disaster Management Guidelines. Preparation of State Disaster Management Plans.

[8] www.ndma.gov.in.

